# The incidence of TB and MDR-TB in pediatrics and therapeutic options: a systematic review

**DOI:** 10.1186/s13643-022-02023-1

**Published:** 2022-08-04

**Authors:** Sheetal Harichander, Ebenezer Wiafe, Kofi Boamah Mensah, Varsha Bangalee, Frasia Oosthuizen

**Affiliations:** 1 Discipline of Pharmaceutical Sciences, College of Health Sciences, University of KwaZulu-Natal, Durban, South Africa; 2 Clinical Pharmacy Services Unit, Directorate of Pharmacy, Ho Teaching Hospital, Ho, Ghana; 3 Department of Pharmacy Practice, Faculty of Pharmacy and Pharmaceutical Sciences, College of Health Sciences, Kwame Nkrumah University of Science and Technology, Kumasi, Ghana

**Keywords:** Tuberculosis, Drug resistance, Multidrug resistance, Pediatrics, Incidence, Treatment, Outcome

## Abstract

**Background:**

Tuberculosis (TB) is considered one of the top 10 causes of death worldwide and the leading cause of death from a single infectious agent. Multidrug-resistant (MDR) TB can affect people of all age groups, including children (aged 0–15 years). However, very little is known about the extent of this problem in children. This systematic review aims to investigate the incidence of TB and drug-resistant (DR) TB among the pediatric population. It also reviews the therapeutic options available to treat the condition.

**Methods:**

A comprehensive search for all relevant evidence was conducted. The following databases were searched: MEDLINE, CINAHL, and Web of Science. The searched time frame was limited from January 1990 to December 2020 with a focus on the incidence of TB and MDR-TB among pediatrics and the therapeutic options available.

**Results:**

A total of 537 articles were obtained via the selected databases. After title and abstract screening, 418 articles were excluded leaving 119 articles. Full-text screening was conducted on 119 articles, excluding a further 110 articles. Thus, 9 articles were subject to quality assessment and included in this review. The 9 articles represented the age group of 0–15 years and included both males and females. All studies included were of retrospective study design.

**Discussion:**

The included studies mentioned a moderate increase in TB cases among pediatrics exacerbated by malnutrition, lack of bacille Calmette-Guérin (BCG) vaccination, and human immunodeficiency virus (HIV) coinfection. MDR-TB prevalence was especially high in South Africa. Drug therapy for both TB and MDR-TB yielded favorable outcomes among pediatrics. However, one of the biggest challenges with drug therapy includes the dosage forms available.

**Systematic review registration:**

DOI: 10.17605/OSF.IO/G34NF

**Supplementary Information:**

The online version contains supplementary material available at 10.1186/s13643-022-02023-1.

## Background

Tuberculosis (TB) is an infectious disease [1]. It is considered one of the top 10 causes of death worldwide and the leading cause of death from a single infectious agent [1]. TB is caused by the bacillus *Mycobacterium tuberculosis*, which is spread when people who are sick with TB expel bacteria into the air; for example, by coughing [1]. The disease naturally affects the lungs (pulmonary TB) but can also affect others parts of the body (extra-pulmonary TB) [1]. Not every individual infected with TB bacteria becomes sick [2]. As a result, two TB-related conditions exist: latent TB infection and TB disease [2]. Latent TB infection occurs when a person has TB bacteria in his or her body, but does not have symptoms of the disease [3]. The infected person’s immune system fights off the TB organisms, and the TB remains inactive throughout life in most people who are infected [3]. This person would have a positive skin test, but a normal chest X-ray [3]. Whereas TB disease is characterized as a person who has signs and symptoms of an active infection [3]. The person would have a positive skin test and a positive chest X-ray [3].

Globally, an estimated 10.0 million people fell ill with TB in 2019 [1]. There were an estimated 1.2 million TB deaths among human immunodeficiency virus (HIV) negative people in 2019 and an additional 208,000 deaths among HIV-positive people [1]. Men (aged ≥ 15 years) reported 56% of the people who developed TB in 2019; women reported for 32% and children (aged < 15 years) for 12% [1].

Drug-resistant (DR) TB represents a major threat to the fight against TB globally [4, 5]. MDR-TB is the most common form of DR-TB defined as a disease caused by strains of *Mycobacterium tuberculosis* resistant to at least isoniazid and rifampicin, the two most powerful first-line anti-tuberculosis drugs [4, 5]. Globally, most cases of MDR remain undetected and untreated because of limited laboratory capacity to conduct tests for drug resistance and limited access to second-line treatment, which is long, toxic and expensive [4–6]. In some cases, even more severe drug-resistant TB may develop [7]. Extensively drug-resistant TB (XDR-TB) is a form of MDR-TB with additional resistance to more anti-TB drugs [7]. As of 2021, the World Health Organization (WHO) has updated its definition for extensively drug-resistant tuberculosis (XDR-TB) and has, for the first time, defined pre-XDR-TB [8]. Pre-XDR-TB is defined as disease caused by *M. tuberculosis* strains that are resistant to isoniazid, rifampicin, and any fluoroquinolone and XDR-TB is now defined as TB caused by *Mycobacterium tuberculosis* strains that are resistant to isoniazid, rifampicin, any fluoroquinolone, and either bedaquiline or linezolid (or both) [8]. Both of these conditions respond to even fewer available medicines [7].

As with other forms of tuberculosis, MDR-TB can affect people of all age groups, including children (aged 0–14 years). However, very little is known about the magnitude of this problem in children, although TB remains one of the top 10 causes of death among children worldwide [6, 9]. The diagnosis of MDR-TB is bacteriological by definition, based on the isolation of strains resistant to medicines [9, 10]. While isolating *M. tuberculosis* in adults with pulmonary tuberculosis is generally an easy procedure (the exception is patients who are immunocompromised), children mainly have paucibacillary disease, which means that specimens for culture and drug susceptibility testing are often difficult to obtain, particularly from the youngest who cannot expectorate sputum [10].

Children with MDR-TB are treated in a parallel way to adults with MDR-TB [9]. One difference is that confirmation and drug susceptibility test (DST) may not possible so that empirical treatment is frequently required for children with suspected MDR-TB. Although outcome data in children are limited, the available evidence suggests that outcomes at least as good as those reported in adults can be achieved [11, 12].

Treating children with MDR tuberculosis is complex [13]. Few of the multiple drugs routinely used to treat MDR tuberculosis have been studied in children, and direction on drug regimens, dosages, appropriate monitoring, and duration of therapy is frequently established from adult data [13]. As young children metabolize drugs more rapidly than adults and generally have paucibacillary disease, this may not always be suitable [13].

### Review question

What is the incidence of TB and MDR-TB in pediatrics and the therapeutic options available to treat the condition?

### Objectives

- To identify the incidence of TB in pediatrics.
- To identify the incidence of MDR-TB in pediatrics.
- To identify the therapeutic options available to treat TB and MDR in pediatrics.
- To identify the therapeutic options available to treat MDR-TB in pediatrics.
- To identify the dosage forms that are currently available for TB and MDR-TB in pediatric patients.

## Methodological considerations

To attain our objectives, the following steps were followed:

1. Development of a literature search strategy to identify the incidence of TB and MDR-TB, the treatment outcomes, and the dosage forms available among pediatrics.
2. Screening of all the identified studies in objectives 1–5 for their relevance in addressing the research objectives.
3. Critical appraisal of the evidence obtained from included studies.
4. Extraction of relevant data from studies on the incidence of TB and MDR-TB and the treatment outcomes among pediatrics

### Search strategy

A comprehensive search for all relevant evidence regardless of language or publication status was conducted. The following databases were searched: MEDLINE, CINAHL, and Web of Science. The search strategy can be found in Additional file 1. The search strategy used a combination of Medical Subject Heading terms and free-text words in titles, abstracts, and keywords. Terms related to TB or MDR-, incidence, drugs, and treatment outcomes were included. The searched time frame was limited from January 1990 to December 2020. SH was credited with the development of the search strategy. EW was credited with the review of the search strategy. Testing of the search strategy was conducted by SH and EW. Following the screening, studies that fulfilled the inclusion criteria and adequately address the research objectives shall be retained for full-text review. The reference lists of included studies and previous reviews will be explored to identify other eligible studies.

### Selection criteria

#### Inclusion criteria

##### Population studied

The study was based on the evaluation of dosage forms available for the treatment of TB and MDR-TB, therefore, included the population from 0 to 15 years of age. Pediatric tuberculosis (TB) represents a major public health concern worldwide [14]. About 1 million children aged less than 15 years develop TB each year, contributing to 3–25% of the total TB caseload [14]. Both male and female pediatric patients will be considered for the review within the age limit as specified for the study. Participants of the study will not be limited to geographical locations or cultural backgrounds.

##### Investigated condition

The review will be assessing the incidence of TB and MDR-TB among the pediatric population.

##### Context of importance

The context includes all studies conducted in pediatric patients globally and is not limited to any cultural backgrounds.

##### Outcome

The outcome of the review is to assess the incidence of TB and MDR-TB among the pediatric population. It will also review the therapeutic options available to treat TB and MDR-TB in terms of the dosage forms available.

##### Types of studies

Studies published between the periods of January 1990 to December 2020 will be considered for the review. This review will only include studies that have been published in the English language. Studies that contain an abstract with a full text available will be considered.

### Exclusion criteria

- Studies that were published before January 1990 or after December 2020.
- Studies that include children above the age of 15 years.
- Studies in which the age of the children studied cannot be determined.
- Studies that include both children and adults.
- Studies that did not indicate the number/percentage of included children.
- Studies that were not published in the English language—for this review, all the authors are only familiar with the English language.
- Studies that do not include the outcome of the review—the outcome is the purpose of the review.
- Studies published without abstracts - abstract assessments are the first step in quality assurance for the review.
- Pilot studies—this type of study is a test study and not an actual study.

SH and EW ran a comprehensive search on the three selected databases. SH merged all the data from the databases to build a library on Endnote® X8.1 library. SH identified and removed any/all duplicates. SH and EW independently assessed the retrieved titles and abstracts of relevant studies for their eligibility to be included in the review. Relevant data will be extracted from each eligible article and retained for full-text review. SH and EW the performed independent screening of the full texts of retained articles with consensus in each stage. A third reviewer, KBM was consulted to resolve any or all disagreements. All studies that met the inclusion criteria were selected. If eligibility was unclear because of missing information, two attempts were made to contact the authors of the primary report. After two unsuccessful attempts, reports were excluded.

The inclusion and exclusion criteria for the study have been presented Table 1. These criteria were defined using the PICOS (Population, Intervention/Investigation, Comparator, Outcome, Study design) approach.

**Table 1:** Selection criteria for studies to be included in systematic review assessing the incidence of TB and MDR-TB and the therapeutic outcomes among paediatrics

Item	Inclusion criteria	Exclusion criteria
**Population**	-Studies involving paediatrics ≤15 years. -Studies including both male and female patients.	Studies involving -Participants ≥15 years -Pregnant participants -Participants with multi-morbidities
**Intervention / Investigated condition**	The review will be assessing the incidence of TB and MDR-TB among the paediatric population and the therapeutic options available.	-Studies describing combined treatment of more than 2 diseases (TB or MDR-TB /HIV)
**Comparison /Context**	-Studies conducted in paediatric patients globally -Studies conducted in paediatric patients of all cultural backgrounds	
**Outcome**	-Studies assessing the incidence of TB and MDR-TB among the paediatric population. -Studies assessing the therapeutic options available to treat TB and MDR-TB in terms of the dosage forms available.	Studies describing outcomes that are unrelated to the incidence and treatment of TB and MDR-TB
**Type of studies**	Cohort studies that use quantitative research methods and Observation studies	-Mini-reviews, conference abstracts, letters to editors, editorials, commentaries. -Studies without an abstract -Studies whose full data will not be available even upon requesting from the author -Duplicates studies: for studies published with the same or different titles or in more than one journal, the most updated version shall be considered.

#### Methodological quality appraisal, data extraction, and cleaning

Crombie’s tool was used to assess the quality of included studies [15]. The methodological quality assessment tool was adopted and modified for this review due to the similarities this review shares with the study conducted by Tola et al. (2020) [16]. This tool contains six items for quality assessment of cross-sectional/prevalence studies. The quality of each article was given by the score ranged from 0 to 6. The quality assessment tool can be found in Additional file 2. Final inclusion of the study was decided through consensus of SH and EW and in the case of disagreement, KBM resolved.

SH and EW completed data extraction and compiled the findings together. KBM addressed any inconsistent data and checked for completeness.

#### Data synthesis and statistical analysis

**Fig. 1:**
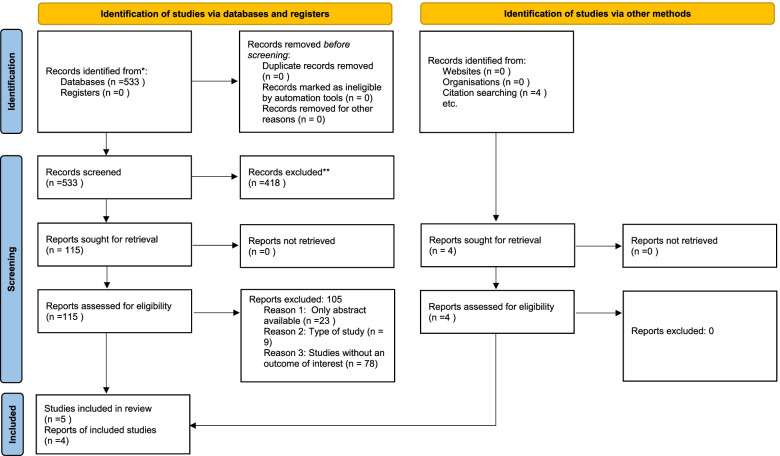
Flow diagram of the study selection process

The review findings were analyzed and compiled in Fig. 1, Tables 2 and 3.

**Table 2:** Characteristics of selected records

Author, year and reference	Research Question(s) or Objective(s)	Research Design	Study Country (Location)	Duration of Study	Sample Size (and Study Population)	Age of Participants (years)	Co-morbidity(ies)
Gledovic et al. 2006 [13]	The objective of this study was to establish the tuberculosis incidence and mortality in children in Serbia in the period 1992-2002	Retrospective	Serbia	1992 - 2002	135000 Male and Female	0-14 years	Missing
Padayatchi et al. 2006 [17]	Investigation into Multidrug-Resistant Tuberculous Meningitis in Children in Durban, South Africa	Retrospective	Durban, South Africa	1992-2003	8 Male and Female	0-11 years	HIV
Fairlie et al. 2011 [18]	The aim of this study was to review the prevalence and clinical outcome of childhood MDR-TB at two hospitals in Johannesburg, South Africa.	Retrospective	Gauteng, South Africa	2008	1317 Male and Female	<14 years	HIV
Seddon et al. 2012 [9]	To describe the clinical presentation, treatment, and outcome for a large cohort of children with confirmed MDR-tuberculosis and evaluate factors influencing treatment response.	Retrospective cohort	Western Cape , South Africa	1^st^ January 2003 to 31^st^ December 2008	111 Male and Female	Median age= 50 months	HIV
Erkens et al. 2014 [19]	The objective of the study was to describe the occurrence of TB events among children in the Netherlands	Retrospective	Netherlands	1993-2012	8357 Male and Female	<14 years	Missing
Nabukeera-Barungi et al. 2014 [20]	Investigation into the presentation and outcome of tuberculous meningitis among children	Retrospective	South Africa	1^st^ January 2009 to 31^st^ December 2009	40 Male and Female	Median age=32 months	HIV
Ben Ayed et al. 2019 [21]	The study aimed to give an update about the epidemiological profile of childhood TB in south of Tunisia and to describe their chronological trends from 1995 to 2016.	Retrospective	Southern Tunisia	1^st^ January 1995 to 31^st^ December 2016	204 Male and Female	<15 years	Missing
Wang et al. 2020 [22]	The study aimed to analyse the epidemiological and clinical characteristics, as well as drug resistance in culture-confirmed children with Tuberculosis meningitis (TBM) in Southwest of China.	Retrospective	Public Health Clinical Center of Chengdu, China	January 2013 and December 2018	319 Male and Female	<14 years	Missing
Zhou et al. 2020 [23]	To evaluate the prevalence of MDR-TB among children age ≤15 years old, from 2008 to 2018.	Retrospective	Shandong Provincial Chest Hospital, Shandong- China	2008 to 2013 and 2014 to 2018. 10 years	222 Male and Female	Mean age of children was 10.7 ± 4.8 years (range from 3 months to 15 years)	Missing

**Table 3:** Summary of findings from selected records

Study Title (Reference)	Quality Appraisal Score (Quality Grade)	Study Findings			Conclusion	Limitations
		Incidence of Paediatric		Paediatric therapeutic options		
		TB	MDR -TB			
Childhood tuberculosis in Serbia. (Gledovic et al. [13]).	4 (Moderate)	1. In the period 1992–2002, 280 Serbian children were reported as having newly diagnosed tuberculosis. 2. 129 were boys and 151 were girls (gender ratio, 0.8:1). 3. The majority of children, 217 (77.5%), were in the age group 5–14 years. 4. The average annual incidence rate in the observed period was 1.79/100,000 (95% confidence interval, 0.92–3.13). 5. The rate for girls was higher than in boys. 6.The rates for both boys and girls were higher in the age group 5–14 years than for the age group 0–4 years	Missing	Missing	In Serbian children, tuberculosis was more frequent in the age 5–14 years (77.5%) than in the youngest age group. This finding is opposite to that of other countries.	1. Retrospective study in nature therefore some data could be missing or incomplete.
Multidrug-Resistant Tuberculous Meningitis in Children in Durban, South Africa. (Padayatchi et al. [17])	5.5 (High)	Missing	1. A retrospective review of medical records of children with culture-confirmed multidrug-resistant tuberculous meningitis (MDR-TBM) at King George V Hospital in Durban, South Africa. 2. Between 1992 and 2003, there were 8 children with MDR-TBM; 6 were HIV infected and 2 were HIV negative. Only one child survived. 3. All the children either had Calmette-Gue´rin bacillus (BCG) scars or a history of BCG vaccination.	Therapeutic options treated with include INH, Rif, PZA, Emb, Eth, Ofloxacin and Streptomycin	Factors that contributed to the high mortality were disseminated TB, HIV infection, delay in diagnosis and treatment, the absence of a standardized approach to the management of MDR-TBM and the poor CSF penetration of most MDR-TB drugs. MDR-TB therapy should be considered if there is a history of TB: a MDR-TB contact or a poor clinical response to TB therapy despite adequate adherence to treatment. Early diagnosis is important because TBM in children is often associated with a serious outcome.	1. The study was conducted in only one Hospital Facility. 2. The study focused on TB meningitis specifically as a form of MDR-TB. 3. Small sample size.
High prevalence of childhood multi-drug resistant tuberculosis in Johannesburg, South Africa: a cross sectional study. (Fairlie et al. [18])	6 (High)	1. 1317 children were treated for tuberculosis in 2008 between the two hospitals where the study was conducted. 2. Drug susceptibility testing was undertaken in 148 (72.5%) of the 204 children who had culture-confirmed tuberculosis. 3. The prevalence of HIV co-infection was 52.1%.	1. The prevalence of isoniazid-resistance was 14.2% (*n* = 21) (95%CI, 9.0-20.9%) and the prevalence of MDR-TB 8.8% (n = 13) (95%CI, 4.8-14.6%). 2. The prevalence of HIV co-infection was 53.9% in children with MDR-TB.	1. Included treatment with pyrazinamide, ethionamide, ethambutol, amikacin and ofloxacin in the intensive phase of treatment with the addition of terizidone and/or kanamycin if additional resistance patterns were present. 2. Continuation phase MDR-TB treatment consisted of ethionamide, ethambutol and ofloxacin	The study demonstrated a high prevalence of drug-resistant MTB in a cohort of children diagnosed with culture-confirmed tuberculosis in Johannesburg, South Africa; this likely represents a large burden of undiagnosed drug-resistant MTB in household and community adult contacts of these children. All child tuberculosis suspects in settings with a high prevalence of tuberculosis and HIV should have confirmation of their HIV infection status. Furthermore, we recommend that routine DST should be performed on MTB isolates obtained from children with culture-confirmed TB in these high-burdened settings.	1. May have overestimated the true MDR-TB prevalence in children in Johannesburg, as patients attending referral hospitals may be at higher risk for drug-resistant tuberculosis compared to those investigated and treated at primary care facilities. 2. Furthermore, DST was performed in only 72.5% of children with culture-confirmed tuberculosis at clinician discretion. 3. Drug susceptibility testing against pyrazinamide is not routinely performed in our setting and is only performed on clinician request. 4. As the study was retrospective and record-based, contact history data may be inaccurate. 5. Detailed data on children with drug-susceptible MTB was not available for this study, as information was collected from laboratory records.
Culture-confirmed multidrug-resistant tuberculosis in children: clinical features, treatment, and outcome. (Seddon et al. [9]).	6 (High)	Missing	1. 111 children with MDR-tuberculosis were identified, with a median age of 50 months. 2. Forty-two samples underwent DST to second-line drugs, which identified 3 MDR-tuberculosis cases resistant to amikacin, 4 resistant to ofloxacin, and 5 resistant to both ofloxacin and amikacin (XDR-tuberculosis). 3.Fifty-three (62%) of 85 children with a sputum result were smear positive; a positive sputum smear was more common in older children (median age, 85 months [IQR, 25–132 months] vs 24 months [IQR, 15–59 months].	The following drugs were used as treatment: - High dose INH - Rif - PZA - Eth - Emb - Streptomycin - Amikacin - Capreomycin - Ofloxacin - Terizadone or cycloserine - *Para*-aminosalicylic acid - *Linezolid*	In conclusion, although South African children with confirmed MDR-tuberculosis often present with severe disease and have a high frequency of HIV infection, excellent treatment outcomes can be achieved in high-burden settings with individualized clinical care under standard programmatic conditions.	1. Reliance on routine data. 2. No systematic data regarding adverse effects, and the tolerability of multiple medications, frequently unpalatable, was not systematically assessed. 3. Although all samples were confirmed to be MDR-tuberculosis, DST for second-line drugs was not routinely completed during the study period. 4. Finally, although treatment outcomes were good, no comment on morbidity as a result of MDR-tuberculosis disease and treatment.
The epidemiology of childhood tuberculosis in the Netherlands: still room for prevention. (Erkens et al. [19])	6 (High)	1. The absolute number of children with TB decreased from 106 in 1993 to 50 in 2012 2. Overall childhood TB incidence has declined over the last two decades from 3.6 in 1993 to 1.9 per 100,000 children in 2012.	Missing	Missing	Children with TB in the Netherlands are generally detected at an early stage and treatment completion rates are high. However, more TB cases among children can be prevented through enhancing TB case finding and screening and preventive treatment of latent TB infection among migrant children, and improving the coverage of BCG vaccination among eligible risk groups.	1. A limitation of the study is that the data are retrieved from the routine TB surveillance registry and are not collected systematically with a strict research design. 2.The Dutch surveillance system is generally regarded as sound and representative of TB incidence. 2.In passively detected TB cases BCG status is not relevant for the clinical management and therefore more likely to be missing. Thus BCG vaccination coverage among the child TB cases may be underestimated.
Presentation and outcome of tuberculous meningitis among children: experiences from a tertiary children's hospital. (Nabukeera-Barungi et al. [20])	3.5 (Moderate)	1. Of 22,943 children admitted to RCWMCH during the study period, we identified 40 children newly diagnosed with TBM; an incidence rate of 1.7 per 1000 admissions. 2. Of the 40 children diagnosed with TBM, 6 (15%) had definitive TBM, 17 (42.5%) had probable TBM and 17 (42.5%) had possible TBM.	Missing	Missing	We found that TBM mainly presented with acute non specific symptoms but the rigorous diagnostics helped make a quick diagnosis and start early treatment. Outcome of treatment at discharge was good with less than 10% mortality and half with neurological sequelae at discharge from hospital. Poor outcome was associated with TBM stage III disease.	1. Retrospective design in which data recording was not standardized and as such some information was missing. 2. Another limitation was with our entry point which was the hospital records department. 3. Some TBM diagnoses may have been missed out just as we found that some codes were in error.
The growing burden of childhood tuberculosis in Southern Tunisia: temporal trends across two decades: 1995-2016. (Ben Ayed et al. [21])	6 (High)	1. Overall, 204 cases of TB were noted in children. 2. The average incidence rate of overall TB was 4.09/100000 population/year. 3. There was a significant rise in extrapulmonary tuberculosis (EPTB) incidence (APC=2.76%; 95% confidence interval (95% CI)=[0.40-5.00]), while pulmonary tuberculosis PTB incidence rate showed a non-significant decrease over time. 4. The under-fives had a significant growing trend (APC of 3.95%; 95% CI=[0.80-7.30]). 5. A significant upward trend of TB incidence in rural districts (APC=4.91%; 95% CI=[1.90-8.10]).	Missing	Missing	The study provided an insight into the burden of childhood TB in South of Tunisia. A significant rise in TB incidence rate was observed among high risk groups. Therefore, implementing preventive and control strategies must be urgently prioritized, with an emphasis on contact screening, maintaining a high BCG vaccination coverage and awareness-raising activities in the community in order to reduce TB transmission in this highly vulnerable population.	1. Firstly, because of the retrospective data collection, the patients were not followed-up during the study period and the treatment outcome as well as the response to therapy could not be assessed. 2. Secondly, analyzing data from a delimited area may not reflect the real burden of childhood TB in the whole country. 3. Another limitation of this study is possible missing or incomplete data, as well as potential biases and errors during date entry.
Epidemiological, clinical characteristics and drug resistance situation of culture-confirmed children TBM in southwest of China: a 6-year retrospective study. (Wang et al. [22])	4.5 (High)	1. Among 319 patients clinically diagnosed with TBM, 42 (13.2%) were Mycobacterial culture positive. 2. Their median age was nine years, and the distribution was equal among female and male patients.	Missing	Missing	TBM among children in Southwest China was mainly concentrated in the minority areas of western Sichuan and more than 95% of patients did not receive BCG vaccination at birth. The most common symptoms were fever, headache, and neck stiffness and all patients had positive chest X-ray findings. In addition, high rates of drug resistance were found.	1. Major limitation was that the study was retrospective in nature.
Prevalence of multidrug-resistant tuberculosis in suspected childhood tuberculosis in Shandong, China: a laboratory-based study. (Zhou et al. [23])	5.5 (High)	Missing	1. In Shandong, the prevalence of MDR-TB in childhood TB was low, at 5.6%. 2.Between 2008-2013 and 2014-2018 among children with TB, the prevalence of MDR-TB remained unchanged, the proportion with pulmonary TB decreased from 78.3% to 64.9%	Missing	The prevalence of MDR-TB among childhood TB in Shandong, China was low and has remained stable over the past years. However, non-tuberculous mycobacterial diseases may be a new challenge in the management of suspected childhood TB.	Some limitations exist. In our study, 12 MDR-TB patients were included, and the average annual number of patients with MDR-TB was fewer than two. If comparing results in each year from 2008 to 2018, statistical significance may never be achieved. For this reason, we divided the study into two periods: 2008 to 2013 and 2014 to 2018.

## Results

The review yielded 533 results. Detailed citation screening resulted in 4 additional studies been added resulting in 537. There were no duplicates. The screening of the articles is depicted in Fig. 1. Title screening led to 418 articles being excluded resulting in 119 remaining for full-text screening. Full-text screening led to 9 articles being selected for the review.

### Characteristics of the included studies

The characteristics of the 9 studies selected are detailed in Table 2. The study years ranged from 2006 to 2020. The studies represent the continents of Africa, Asia, and Europe. No studies were conducted in Australia, the North and South of America. All studies included both male and female participants. The general ages represented include 0 to 15 years. All studies were of the retrospective design. Four studies included participants with HIV co-infection. Of the 9 studies, the following variations of TB were mentioned, i.e.: TB, PTB, EPTB MDR-TB, TBM, and isoniazid-resistant tuberculosis.

### Quality assessment of the included studies

According to the scoring tool, only 2 studies were of moderate quality and 7 studies were of high quality (Additional file 1). No studies were excluded based on the quality assessment tool. There was no disagreement between SH and EW.

### Review findings

The summary of the findings of the studies included is detailed in Table 3.

Of the 9 studies, 5 studies focused on TB (EPTB, PTB, and TBM). Only Erkens et al. (2014) [20] study reported a significant reduction in the TB statistics whereas the other studies noted slight increases that occurred predominantly in the age group 5–14 years. The majority of studies noted an equal distribution of the condition among female and male participants.

Four articles mentioned MDR-TB. Three of these studies were conducted in South Africa. Although MDR-TB is growing in South Africa among the adult population, the statistics remain relatively low in the pediatric population but higher in South Africa in comparison to China [10, 18, 24, 25]. Zhou et al. (2020) mentioned that in Shandong, the prevalence of MDR-TB in childhood TB was low, at 5.6% [25].

Only three articles stated the therapeutic options available for the treatment of TB and DR-TB. The following drugs were mentioned: rifampicin (Rif), isoniazid (INH), pyrazinamide (PZA), ethambutol (Emb), ethionamide (Eth), ofloxacin, streptomycin, amikacin, terizadone, kanamycin, capreomycin, cycloserine, *para*-aminosalicylic acid, and linezolid. Padayatchi et al. reported only 1 favorable outcome and 7 deaths among the participants [13]. Fairlie et al. (2011) and Seddon et al. (2012) reported 76.9% and 82% favourable treatment outcomes [8, 17]. Therefore, children with MDR tuberculosis can be treated successfully.

In this data set, children infected with HIV were more likely to have confirmed MDR-TB. Three of the four studies were conducted in South Africa. South Africa has the biggest HIV epidemic in the world [25]. The HIV epidemic in southern Africa has exacerbated the spread and virulence of DR-TB [26]. On the other hand, malnutrition and TB are frequently associated conditions in children, and both represent a public health issue worldwide [26].

## Limitations

### Limitations of the review

- The restriction of the literature search timeline to the range of January 1990 to December 2020.Inclusion of studies published only in the English language.
- Limiting the literature search to three databases.
- All included studies were of retrospective design; hence, there could be missing or incomplete information.
- The review did not have a representative of studies conducted in North America, South America and Australia.

The above limitations can contribute to selection bias and have an impact on the results of this review.

## Discussion

Childhood TB adds nearly 15–20% of all TB cases worldwide [25–27]. TB can imitate signs and symptoms of many common childhood diseases, including pneumonia, malnutrition, and HIV infection which pose diagnostic difficulties. Childhood TB has increasingly been recognized as contributing a substantial proportion of the global TB caseload; however, pioneering work has been done spanning diagnosis, prevention, management, and the impact of HIV [28]. This is evidenced by the moderate increases in the condition over the years.

One of the contributing factors to an increase in pediatric TB is the subject of household contact. According to the published literature, a single pulmonary TB (PTB) patient can infect 10 to 15 persons on average [29], having close contact, within a community whereas 90% of the TB transmission in the community is due to sputum smear-positive [30, 31]. Household contacts are highly susceptible to acquire TB infection from the index cases because of their proximity. The goal of contact tracing and their screening for TB could lead to the detection of additional cases of TB, maximizing the impact of case detection and effective treatment [32, 33]. The prevalence of TB disease is particularly soaring among children who are close contacts of a TB patient. Hence, screening children as contacts are generally recommended, though practiced in rarity [34].

Malnutrition is highly prevalent in children living in tuberculosis endemic countries and contributes to 2.2 million deaths in children under 5 years of age globally [35]. Poverty, overcrowding, food insecurity, and human immunodeficiency virus (HIV) further set the stage for both malnutrition and poor infection control [36].

Several studies conducted in Europe noted that an increase in immigration of people from high TB incidence areas has recently contributed to the resurgence of this disease in these countries [24, 37]. Many countries have developed immigration TB screening programs to suit the needs of adults, but have not focused much attention on migrant children [38].

Around 3% of children with TB have MDR-TB, amounting to between 25,000 and 32,000 children developing MDR-TB disease each year [39]. Only 3–4% of them are diagnosed and treated and, as a result, around 21% of children with MDR-TB likely die [39]. Increased implementation of household contact investigations could help to close the treatment gap [39]. According to the included studies in this review, South Africa represented the highest incidence of MDR-TB among the pediatric population.

Diagnosing MDR-TB is especially problematic in children, from whom it is difficult to isolate a bacteriologic specimen that can be used to directly detect drug resistance [40].

Seddon et al. (2014) conducted a cohort study in children in South Africa [41]. In this cohort of children with either bacteriologically confirmed and/or clinically diagnosed disease, treatment was well tolerated overall with few significant adverse events. Treatment outcomes were excellent, with over 90% of children cured or probably cured. Many children were identified and started on treatment early, following the diagnosis of an MDR-TB source case, illustrating the importance of contact tracing. The three children who died either presented late with severe TB disease and concomitant HIV infection or had the extensive disease and had defaulted care [41]. This is supportive of other literature and the studies included in this review. One of the major challenges with drug therapy is very few drugs are produced in pediatric formulations, and the pharmacokinetics are incompletely studied in children [42]. This means that optimal dosing of second-line drugs is unknown and that tablets must be broken or cut, potentially leading to inaccurate dosages and blood concentrations that are sub-therapeutic or toxic [42]. The taste of medications is often unpalatable and a number of the drugs can cause vomiting and diarrhoea. This may affect the amount of the drug absorbed [42].

## Conclusion

A large percentage of pediatric TB and/DR-TB cases remained undetected, or not reported; however, in recent years, a growing number of children are contracting DR-TB, mainly through household transmission. It is critical when adults test positive for DR-TB, that the entire household is screened. Diagnosis of DR-TB is more difficult in children as the disease in children is generally pauci-bacillary. The sputum is rarely produced resulting in the specimens being hard to obtain. The diagnosis in most of cases is clinical, and only rarely bacteriologically confirmed. The treatment of DR-TB in children is guided by the same principles and uses the same second-line drugs as the treatment in adults, although optimal durations of regimens are not known. Children face challenges to appropriate treatment. Not only because of the difficulty in swelling pills, but also because of the challenges to achieve the right dosage and blood concentration, to obtain efficacy of the therapy and to avoid toxicity. With increased MDR-TB rates evident especially in high HIV prevalence areas such as South Africa in this study, infection control needs to be addressed urgently. More data on morbidity and long-term mortality in HIV-infected and HIV-uninfected children, are needed. Knowing the TB burden in the pediatric population, globally and at country level, is fundamental and developing more child-friendly formulations to treat DR-TB would be ideal in combating DR-TB.

## Recommendations

The therapeutic management of children with MDR- andXDR-TB is complicated by a lack of knowledge and the fact that many potentially suitable drugs are not registered for pediatric use and there are no formulations suitable for children. This review highlights the need for more research to be done to assess the incidence and prevalence of DR-TB in pediatrics and the possible demand for the availability of suitable dosage forms to treat the condition.

## Supplementary Information

**Additional file 1.** Search strategy.

**Additional file 2.** Quality assessment tool.

## Data Availability

Data and materials are available from the corresponding author.

## Abbreviations

BCG: Bacille Calmette-Guérin
DR: Drug-resistant
DST: Drug susceptibility test
Emb: Ethambutol
EPTB: Extrapulmonary tuberculosis
Eth: Ethionamide
HIV: Human immunodeficiency virus
INH: Isoniazid
MDR-TB: Multi-drug-resistant tuberculosis
MTB: Mycobacterium tuberculosis
PTB: Pulmonary tuberculosis
PZA: Pyrazinamide
Rif: Rifampicin
TB: Tuberculosis
TBM: Tuberculosis meningitis
XDR-TB: Extensively drug-resistant tuberculosis
