# Correction: The incidence of TB and MDR-TB in pediatrics and therapeutic options: a systematic review

**DOI:** 10.1186/s13643-022-02101-4

**Published:** 2022-10-22

**Authors:** Sheetal Harichander, Ebenezer Wiafe, Kofi Boamah Mensah, Varsha Bangalee, Frasia Oosthuizen

**Affiliations:** 1 Discipline of Pharmaceutical Sciences, College of Health Sciences, University of KwaZulu-Natal, Durban, South Africa; 2 Clinical Pharmacy Services Unit, Directorate of Pharmacy, Ho Teaching Hospital, Ho, Ghana; 3 Department of Pharmacy Practice, Faculty of Pharmacy and Pharmaceutical Sciences, College of Health Sciences, Kwame Nkrumah University of Science and Technology, Kumasi, Ghana

**Correction: Syst Rev 11, 157 (2022)**

10.1186/s13643-022-02023-1

Following publication of the original article [1], the authors identified an error in the published version.

There is an error on the publication with respect to the affiliation of authors with Kofi Boamah Mensah & Frasia Oosthuizen. Affiliation 3 should be affiliated to Kofi Boamah Mensah.

The mistakes were rectified in this article and in the original article.

## References

[1] Harichander S, Wiafe E, Mensah KB (2022). The incidence of TB and MDR-TB in pediatrics and therapeutic options: a systematic review. Syst Rev.

