# Supplementary material for: The incidence of TB and MDR-TB in pediatrics and therapeutic options: a systematic review
Source: Syst Rev. 2022 Aug 4;11:157. doi: 10.1186/s13643-022-02023-1 (PMC9354367; doi:10.1186/s13643-022-02023-1)
Supplement: Supplementary file 1 — Additional file 1. Search strategy. [file 13643_2022_2023_MOESM1_ESM.docx]

An investigation into the incidence of TB, MDR-TB and the therapeutic options available in paediatrics: A systematic review.

| **SN** | **SEARCH TERM** | **RESULTS^a^** | **RESULTS^b^** |
| --- | --- | --- | --- |
| S1 | Incidence OR Frequency OR Incidence rate OR Occurrence OR Prevalence OR Epidemiology OR Frequency OR Statistics | 8,654 | 150,815 |
| S2 | Tuberculosis* OR Mycobacterium tuberculosis OR TB* | 1,193 | 47,550 |
| S3 | Drug resistant* OR Multidrug resistant* OR Multidrug-resistant* OR Multi drug resistant* OR MDR* | 52 | 10,157 |
| S4 | Management options OR Therapeutic options OR Treatment options OR Intervention OR Therapy OR Rehabilitation OR Efficacious options OR Effective approaches | 8,479 | 170,232 |
| S5 | Paediatrics* OR Pediatric* OR Children* OR Child* OR Infant* OR Young person* | 6,394 | 93,540 |
| S6 | S2 AND S3 | 2 | 249 |
| S7 | S2 OR S6 | 82 | 3,158 |
| S8 | S1 AND S7 | 42 | 1,137 |
| S9 | S4 AND S7 | 47 | 1,603 |
| S10 | S8 OR S9 | 66 | 2,107 |
| S11 | S5 AND S10 | 6 | 402 |

**Additional file 1**

***Search Strategy for CINAHL^a^ and Medline^b^ via EBSCOHOST***

*1 JUNE 2021 (between 1:25 GMT – 1:35 GMT)^a^ and (between 1:40 GMT – 2:00 GMT)^b^*

| **SN** | **SEARCH TERM** | **RESULTS** |
| --- | --- | --- |
| #1 | AK=(Incidence OR Frequency OR Incidence rate OR Occurrence OR Prevalence OR Epidemiology OR Frequency OR Statistics) | 277,032 |
| #2 | AK=(Tuberculosis* OR Mycobacterium tuberculosis) | 32,396 |
| #3 | AK=(Drug resistant* OR Multidrug resistant* OR Multidrug-resistant* OR Multi drug resistant* OR MDR*) | 9,843 |
| #4 | AK=(Management options OR Therapeutic options OR Treatment options OR Intervention OR Therapy OR Rehabilitation OR Efficacious options OR Effective approaches) | 295,411 |
| #5 | AK=(Paediatrics* OR Pediatric* OR Children* OR Child* OR Infant* OR Young person*) | 291,137 |
| #6 | #3 AND #2 | 1,687 |
| #7 | #6 OR #2 | 32,396 |
| #8 | #7 AND #1 | 1,599 |
| #9 | #7 AND #4 | 980 |
| #10 | #9 OR #8 | 2,534 |
| #11 | #10 AND #5 | 125 |

***Search Strategy for Web of Science***

*1 JUNE 2021 (between 2:15 GMT – 3:27 GMT)*
