# Supplementary material for: The incidence of TB and MDR-TB in pediatrics and therapeutic options: a systematic review
Source: Syst Rev. 2022 Aug 4;11:157. doi: 10.1186/s13643-022-02023-1 (PMC9354367; doi:10.1186/s13643-022-02023-1)
Supplement: Supplementary file 2 — Additional file 2. Quality assessment tool. [file 13643_2022_2023_MOESM2_ESM.docx]

**Additional file 2**

**APPRAISAL OF METHODOLOGICAL QUALITY OF STUDIES**

| **AUTHOR, YEAR [REFERENCE]** | **QUALITY ASSESSMENT CRITERIA** | | | | | | **TOTAL SCORE (QUALITY GRADE)** |
| --- | --- | --- | --- | --- | --- | --- | --- |
|  | **A** | **B** | **C** | **D** | **E** | **F** |  |
| Gledovic et al.  2006 | 1 | 1 | 0.5 | 0.5 | 0.5 | 0.5 | 4 (MQ) |
| Padayatchi et al.  2006 | 0.5 | 1 | 1 | 1 | 1 | 1 | 5.5 (HQ) |
| Fairlie et al.  2011 | 1 | 1 | 1 | 1 | 1 | 1 | 6 (HQ) |
| Seddon et al.  2012 | 1 | 1 | 1 | 1 | 1 | 1 | 6 (HQ) |
| Erkens et al.  2014 | 1 | 1 | 1 | 1 | 1 | 1 | 6 (HQ) |
| [Nabukeera-Barungi](https://www.ncbi.nlm.nih.gov/pubmed/?term=Nabukeera-Barungi%20N%5BAuthor%5D&cauthor=true&cauthor_uid=26060471) et al.  2014 | 0.5 | 0.5 | 1 | 0.5 | 0.5 | 0.5 | 3.5 (MQ) |
| Ben Ayed et al.  2019 | 1 | 1 | 1 | 1 | 1 | 1 | 6 (HQ) |
| Wang et al.  2020 | 1 | 1 | 1 | 1 | 0.5 | 1 | 5.5 (HQ) |
| Zhou et al.  2020 | 1 | 1 | 1 | 1 | 1 | 1 | 6 (HQ) |

**QUALITY ASSESSMENT CRITERIA**

A= Appropriateness of design to meet the aims; B= Adequate description of the data; C= Adequate representativeness of the sample to total; D= Clearly stated aims and likelihood of reliable and valid measurements; E= Assessment of statistical significance; F= Adequate description of statistical methods.

**SCORE ALLOCATION AND INTERPRETATION**

Mark allocation: 1 = Yes/Reported; 0.5 = Unclear; 0= No

Quality Grade: 0 – 0.33 = Poor Quality (PQ); 0.34 – 0.66 = Moderate Quality (MQ); 0.67 – 1.00 = High Quality (HQ)

Total score = 6
